# RIOK1 kinase activity is required for cell survival irrespective of *MTAP* status

**DOI:** 10.18632/oncotarget.25586

**Published:** 2018-06-19

**Authors:** Alexandra Hörmann, Barbara Hopfgartner, Thomas Köcher, Maja Corcokovic, Teresa Krammer, Christoph Reiser, Gerd Bader, Junwei Shi, Katharina Ehrenhöfer, Simon Wöhrle, Norbert Schweifer, Christopher R. Vakoc, Norbert Kraut, Mark Pearson, Mark Petronczki, Ralph A. Neumüller

**Affiliations:** ^1^ Boehringer Ingelheim RCV GmbH & Co KG, 1120 Vienna, Austria; ^2^ Vienna Biocenter Core Facilities GmbH, 1030 Vienna, Austria; ^3^ Cold Spring Harbor Laboratory, Cold Spring Harbor, NY 11724, USA

**Keywords:** RIOK1, MTAP, PRMT5, cancer, target

## Abstract

Genotype specific vulnerabilities of cancer cells constitute a promising strategy for the development of new therapeutics. Deletions of non-essential genes in tumors can generate unique vulnerabilities which could be exploited therapeutically. The *MTAP* gene is recurrently deleted in human cancers because of its chromosomal proximity to the tumor suppressor gene *CDKN2A*. Recent studies have uncovered an increased dependency of *MTAP*-deleted cancer cells on the function of a PRMT5 containing complex, including WDR77, PRMT5 and the kinase RIOK1. As RIOK1 kinase activity constitutes a potential therapeutic target, we wanted to test if *MTAP* deletion confers increased sensitivity to RIOK1 inhibition. Using CRISPR/Cas9-mediated genome engineering we generated analog sensitive alleles of *RIOK1* in isogenic cell lines differing only by *MTAP* status. While we were able to independently confirm an increased dependency of *MTAP*-deleted cells on PRMT5, we did not detect a differential requirement for RIOK1 kinase activity between *MTAP*-proficient and deficient cells. Our results reveal that the kinase activity of RIOK1 is required for the survival of cancer cell lines irrespective of their *MTAP* status and cast doubt on the therapeutic exploitability of RIOK1 in the context of *MTAP*-deleted cancers.

## INTRODUCTION

The concept of synthetic lethality has emerged as an attractive strategy for the development of targeted cancer therapeutics [1]. In the course of oncogenesis, cancer cell specific vulnerabilities can be generated by the deletion or mutation of driver or passenger genes. This concept has been harnessed in cancer therapy by the development of PARP inhibitors that selectively inhibit survival of cancer cells carrying mutations in homologous recombination repair genes, such as *BRCA1* or *BRCA2* [2]. In addition, several examples for passenger deletion-induced vulnerabilities have been published, including enolase 1/2 in glioblastoma [3] or malic enzyme 2/3 in pancreatic cancer [4].

Three recent studies have reported an increased dependency of cancer cells harboring a homozygous deletion of the *MTAP* gene [5–7] on PRMT5, WDR77 and RIOK1, all members of a PRMT5 containing complex, and the upstream component MAT2A [8–12]. *MTAP* resides in close proximity to the *CDKN2A* locus that encodes the key tumor suppressor proteins p16 and p14 and is frequently co-deleted across a wide range of cancer indications [5–7]. *MTAP* encodes the enzyme S-methyl-5’-thioadenosine phosphorylase that catalyzes the reversible phosphorylation of S-methyl-5’-thioadenosine (MTA) to adenine and 5-methylthioribose-1-phosphate which constitutes a key step in the methionine salvage pathway. Consistently, the bulk levels of the MTAP substrate metabolite MTA are elevated in *MTAP*-deficient cells [5–7].

PRMT5 is the major methyltransferase for mono- and symmetric arginine di-methylation of histone and non-histone proteins. PRMT5 associates with WDR77 (MEP50) and a range of other factors, including RIOK1 and plCln that modulate its substrate specificity. PRMT5 regulates a diverse set of cellular processes, consistent with the wide range of direct methylation targets [13–15]. Although PRMT5 and several of its binding partners are suggested to be core essential genes, required for general cell survival [13–15], it has been proposed that an inhibitory effect of MTA on PRMT5 underlies the increased dependency of *MTAP*-deficient cells on PRMT5 activity. Increased MTA accumulation in *MTAP*-deleted cells has been demonstrated to be associated with a partial inhibition of PRMT5 in a SAM-competitive manner [5–7]. This partial inhibition of PRMT5 upon *MTAP* deletion renders the cells sensitive to further down regulation or inhibition of PRMT5 and its binding partners that are required for efficient methylation.

Here we asked if the kinase activity of RIOK1 is therapeutic target in *MTAP*-deficient cells. Using CRISPR/Cas9 mediated genome engineering [16] we generated analog sensitive alleles [17] of RIOK1 in *MTAP* isogenic cell lines. Using pharmacological inhibition of RIOK1 analog sensitive versions, we found that *MTAP-*proficient and deficient cells depend equally on RIOK1 kinase activity, arguing against the notion that the inhibition of RIOK1 kinase activity can be therapeutically exploited for selectively targeting *MTAP*-deleted cancers. A CRISPR based analysis of PRMT5 and RIOK1 requirement in isogenic cell lines further suggests that the therapeutic window for inhibition in *MTAP*-deficient tumors might be narrow at best, raising doubts regarding the therapeutic exploitability of this therapeutic concept.

## RESULTS

### Generation of MTAP isogenic cells

**Figure 1:**
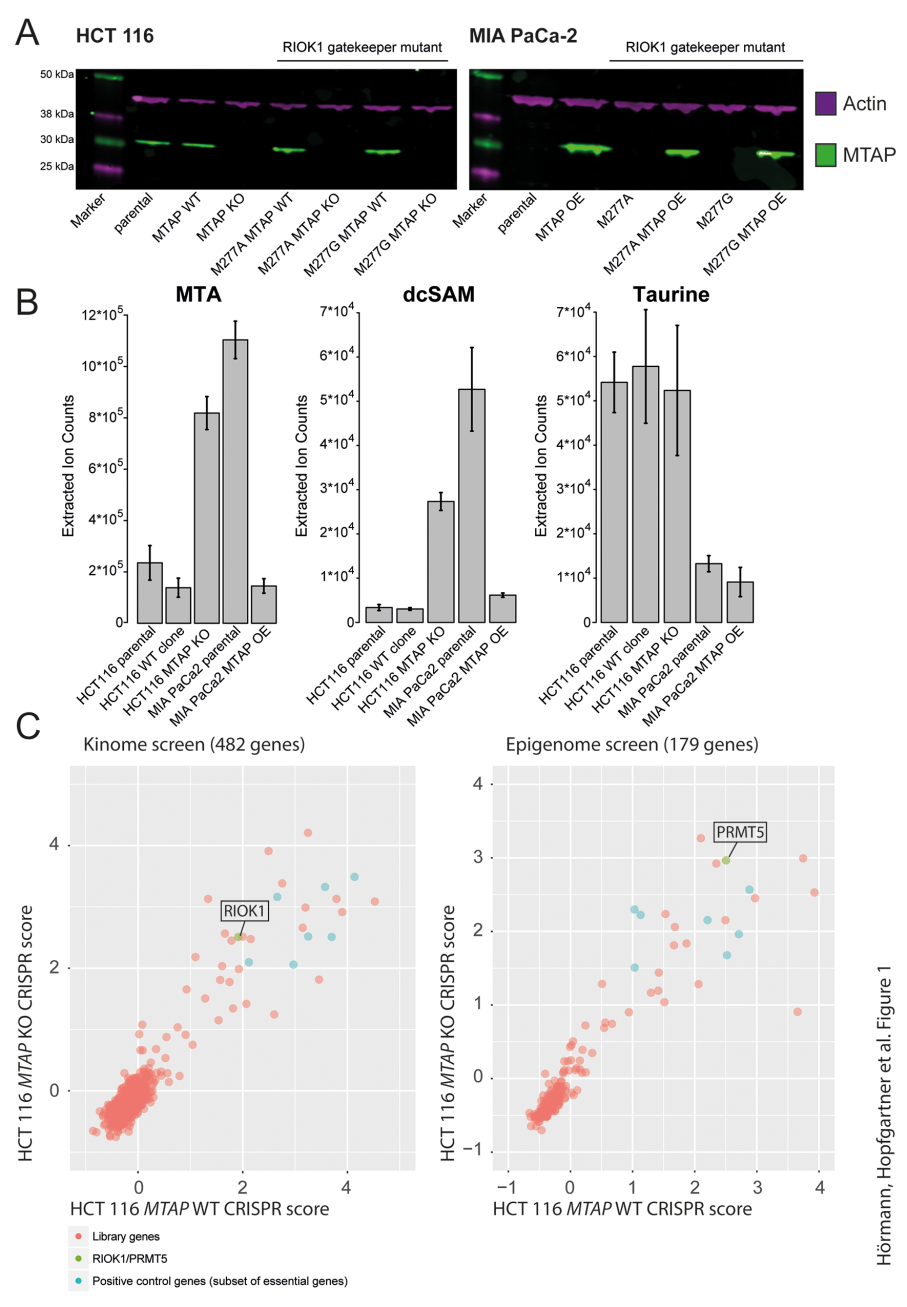
Generation of *MTAP* isogenic cell lines **(A)** Western Blot confirmation of *MTAP* status in HCT 116 and MIA PaCa-2 MTAP isogenic cell lines with and without the RIOK1 gatekeeper mutations M277A and M277G. Green: MTAP; Magenta: Actin loading control. MTAP KO refers to *MTAP* knockout clones; MTAP OE refers to *MTAP* overexpressing cell lines. **(B)** Mass Spectrometry based analysis of a select set of metabolites confirms increased MTA levels upon loss of *MTAP*. Similarly, dcSAM (decarboxylated S-Adenosine Monophosphate) levels correlate with the *MTAP* status, whereas control metabolite (Taurine) levels are not dependent on the *MTAP* status. Bars represent mean and error bars depict the standard deviation. **(C)** Kinome and epigenome CRISPR screens in *MTAP* isogenic cell lines identify *RIOK1* and *PRMT5* as essential genes irrespective of the *MTAP* status. CRISPR scores associated with all screened genes are listed in Supplementary Tables 2–5.

To address the differential requirement of the PRMT5 complex members in *MTAP*-deficient and proficient cells, we generated isogenic cell pairs that differ only in the presence or absence of functional MTAP protein. A similar strategy was employed by previous studies reporting on the increased dependency of MTAP deficient cells on the PRMT5 complex [6, 7]. Using a CRISPR/Cas9-based gene inactivation [16], we generated *MTAP* mutant and wild type cell line clones from the diploid colorectal cancer cell line HCT 116. Probing lysates of these mutant cell lines with a polyclonal antibody raised against MTAP revealed the absence of MTAP protein in the selected knockout (KO) clones, when compared to the parental cell line or MTAP wild-type clones (Figure 1A). Consistent with previous reports [5–7], mass spectrometry analyses detected elevated levels of the upstream metabolites S-methyl-5’-thioadenosine (MTA) and decarboxylated S-adenosylmethionine (dcSAM) in *MTAP* KO cell lines, compared to wild type clones or the parental cell line (Figure 1B). Other metabolites, such as taurine, were measured as internal standards and did not change significantly (Figure 1B). Altogether, these data demonstrate that we have successfully generated isogenic HCT 116 cell lines differing in the functional status of MTAP.

As a parallel strategy, we aimed to reconstitute MTAP expression in an *MTAP*-deficient cell line. We therefore stably expressed MTAP in *MTAP*-deficient pancreatic cancer MIA PaCa-2 cells that contain a homozygous deletion of the *CDKN2A* locus. Western Blot analysis confirmed the efficient introduction of MTAP (OE) (Figure 1A). In agreement with the expression data, reintroduction of MTAP leads to a corresponding decrease in the upstream metabolites MTA and dcSAM in MIA PaCa-2 cells (Figure 1B).

### CRISPR screens reveal no differential sensitivity of *MTAP* isogenic cells

In a next step, we wanted to use a genetic approach to test the increased dependency of *MTAP*-deficient cells on PRMT5 and RIOK1 function. A domain-directed CRISPR/Cas9 strategy [18] was used to assess if PRMT5 and RIOK1 are differentially required in the presence and absence of *MTAP*. A library of >3000 gRNAs targeting 482 human kinases, including *RIOK1* and a library consisting of >1300 gRNAs targeting 179 epigenetic regulators, including *PRMT5* (Figure 1C), were introduced into HCT 116 *MTAP* isogenic cell lines that had been engineered to express Cas9. Consistent with previous findings [13, 15], gRNAs targeting *PRMT5* and *RIOK1* were reduced to a similar extent over time in both *MTAP*-proficient and deficient cells underlining their role as core essential genes (Figure 1C). While our manuscript was under revision, another study independently confirmed that RIOK1 is required for the proliferation of HCT 116 cells [19]. Additionally, no differential sensitivities were observed in the *MTAP* isogenic cell lines in our screens.

**Figure 2:**
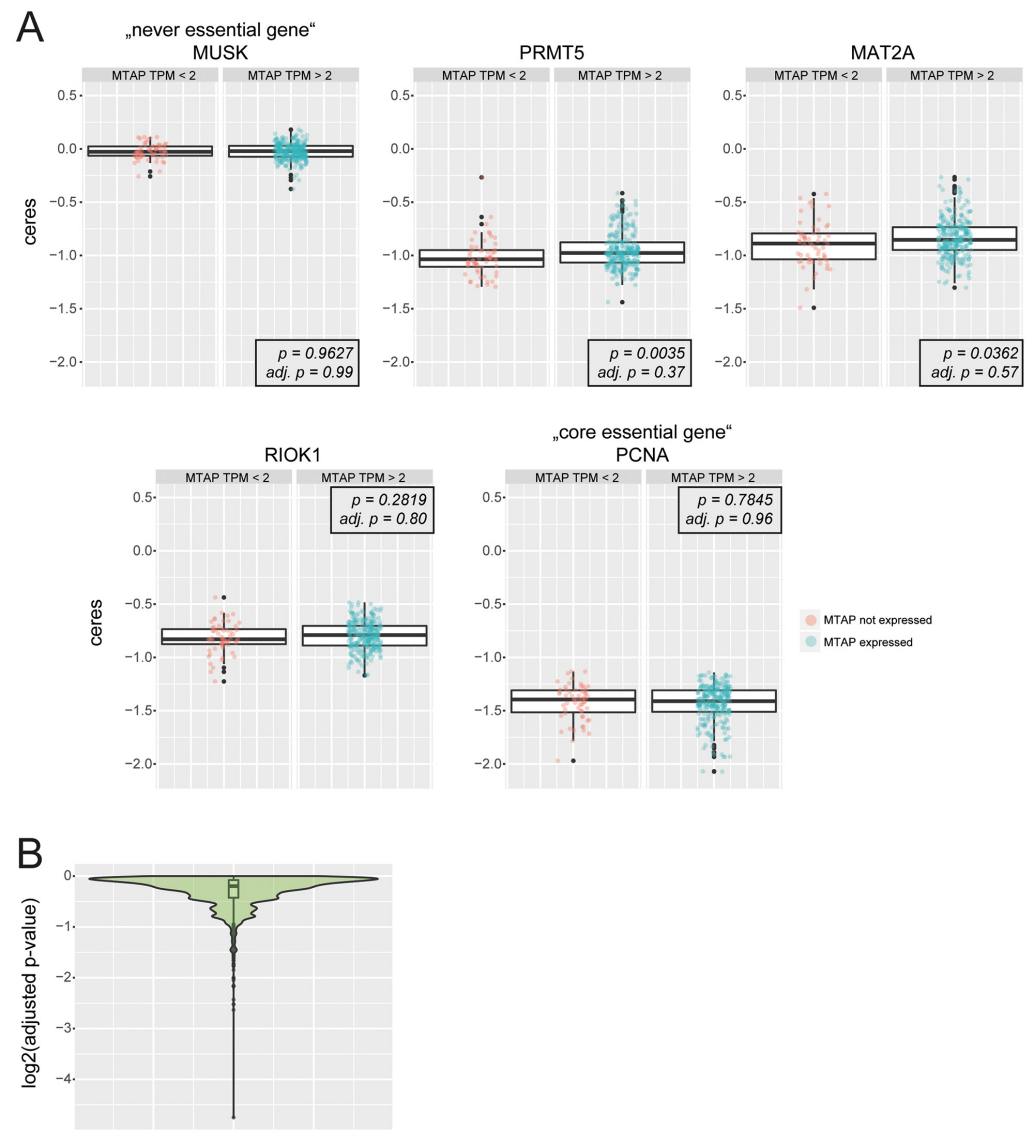
Bio-informatics analysis of publicly available genome-scale CRISPR screening data **(A)** Boxplots (overlaid: individual data points) depict depletion scores between MTAP non-expressing cells (MTAP TPM < 2) and MTAP expressing cells (MTAP TPM > 2) for the “never essential gene” *MUSK*, *PRMT5*, *MAT2A*, *RIOK1* and the “core essential gene” *PCNA*. p-values (*p*) and adjusted p-values (*adj. p*) for Wilcoxon test statistics are indicated. Y- axis depicts the *ceres* scores as reported in [20]. No statistically significant differences between MTAP expressing and non-expressing cells were observed after correcting for multiple testing (*adj. p*). **(B)** Explorative analysis for differentially required genes between MTAP expressing and non-expressing cells. Violin- and boxlot of log2 adjusted p-values (Wilcoxon test).

To corroborate these findings over a larger panel of cells we analyzed publicly available genome-scale CRISPR screening data [20]. We grouped the 342 cell lines screened in this study into MTAP non-expressing (Transcripts Per Million (TPM) < 2) and MTAP expressing (TPM > 2) cells and subsequently performed a Wilcoxon test-based statistical analysis to determine if MTAP expressing and non-expressing cells differ in their sensitivity towards the loss of individual genes (Figure 2A, 2B). A global analysis of all screened genes revealed no differential sensitivities after p-value correction for multiple testing (Figure 2B). gRNAs targeting PRMT5, MAT2A and RIOK1 result in comparable depletion scores between MTAP expressing and non-expressing cells (Figure 2A). To validate the approach we applied the same analysis pipeline to a large-scale RNAi-based loss of function screening resource (DRIVE) [21] that was used by Mavrakis et al. [7] to propose the differential requirement of PRMT5, WDR77, MAT2A and RIOK1 between *MTAP*-proficient and deficient cells. Similar to the findings reported by Mavrakis et al., our analysis revealed a statistically significant, differential requirement of PRMT5, WDR77 and MAT2A between MTAP expressing and non-expressing cells (Supplementary Figure 1A, 1B). Only a modest differential requirement was observed for RIOK1. In addition, it is worth noting that the magnitude of depletion is stronger for PRMT5 and MAT2A in this dataset, raising doubts about the knock-down efficiency of RIOK1 and WDR77 in the DRIVE data [21]. Altogether, our bio-informatics analysis suggests that a pronounced discrepancy exists between the complementary [22] loss of function strategies RNAi and CRISPR with respect to the requirement of PRMT5, MAT2A, WDR77 and RIOK1 in *MTAP* proficient and deficient cells. This discrepancy could stem from differences between hypomorphic versus amorphic phenotypes induced by RNAi and CRISPR, respectively. In order to unambiguously clarify if PRMT5, MAT2A and RIOK1 are differentially required between *MTAP*-proficient and deficient cells, we proceeded to use i) time resolved CRISPR depletion assays and ii) pharmacological inhibition of PRMT5 and RIOK1 as described below.

### Time resolved CRISPR depletion assays

**Figure 3:**
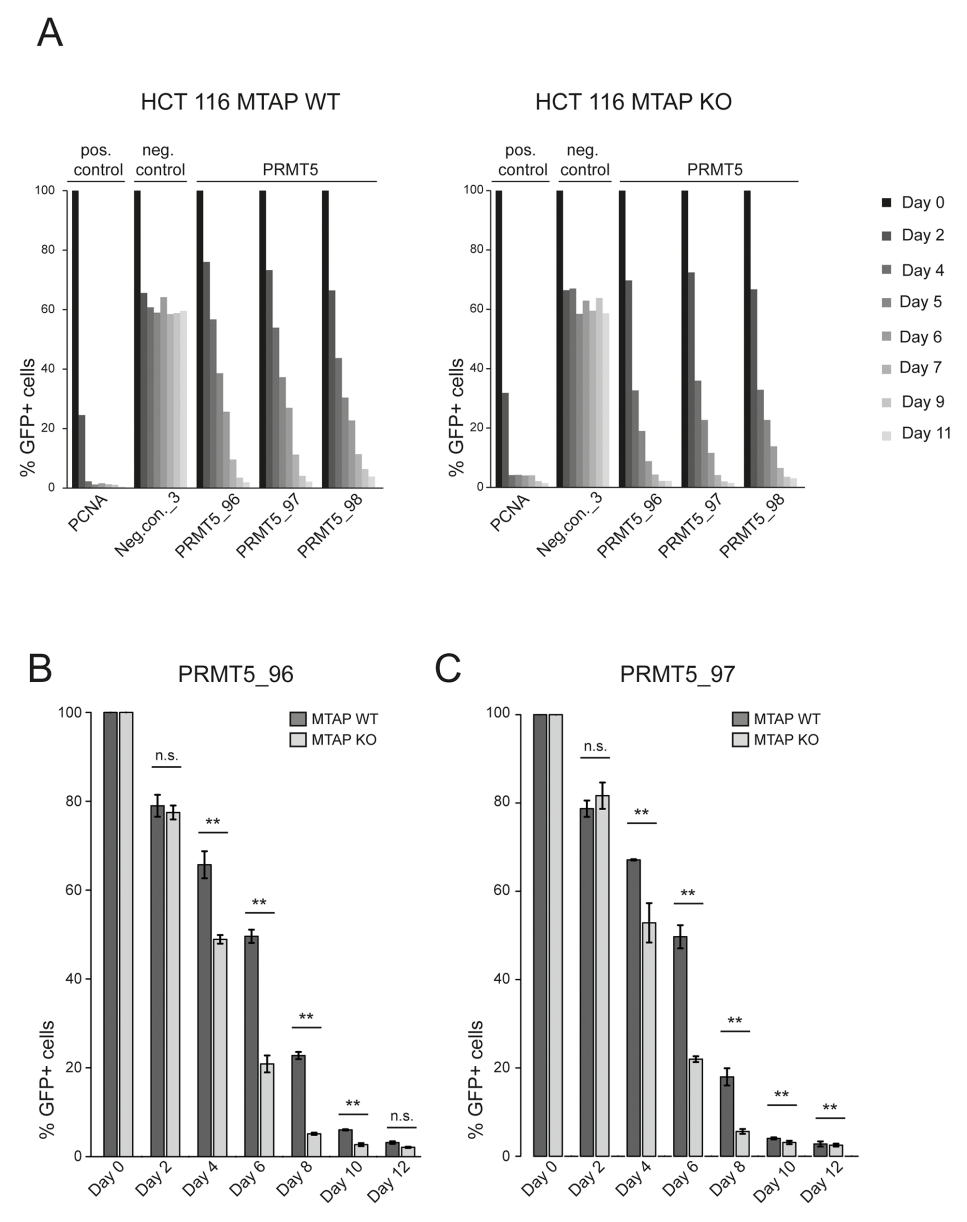
A CRISPR depletion assay confirms the differential requirement of PRMT5 in MTAP isogenic HCT 116 cells **(A)** A timecourse CRISPR depletion experiment, following the depletion kinetics of GFP+ cells (Cas 9 and gRNA expressing) relative to the GFP- cells (Cas 9 expressing) in HCT 116 *MTAP*-proficient (WT) and deficient (KO) cells. *PCNA* serves as a core essential control gene. Neg.con._3 depicts a non-targeting control and PRMT5_96, PRMT5_97 and PRMT5_98, are PRMT5 specific gRNAs. **(B)** The PRMT5 specific gRNA PRMT5_96 results in a faster depletion of GFP+ cells in *MTAP*-deleted (KO) HCT 116 cells compared to *MTAP* WT HCT 116 cells. Bars represent the mean percentage of depletion. Error bars represent the standard deviation (n.s. indicates non-significant; ^**^ denotes p-values < 0.05; *t*-test). **(C)** The PRMT5 specific gRNA PRMT5_97 results in a faster depletion of GFP+ cells in *MTAP*-deleted HCT 116 cells compared to *MTAP* WT HCT 116 cells. Statistical tests and labels are similar to Figure 3 panel B.

**Figure 4:**
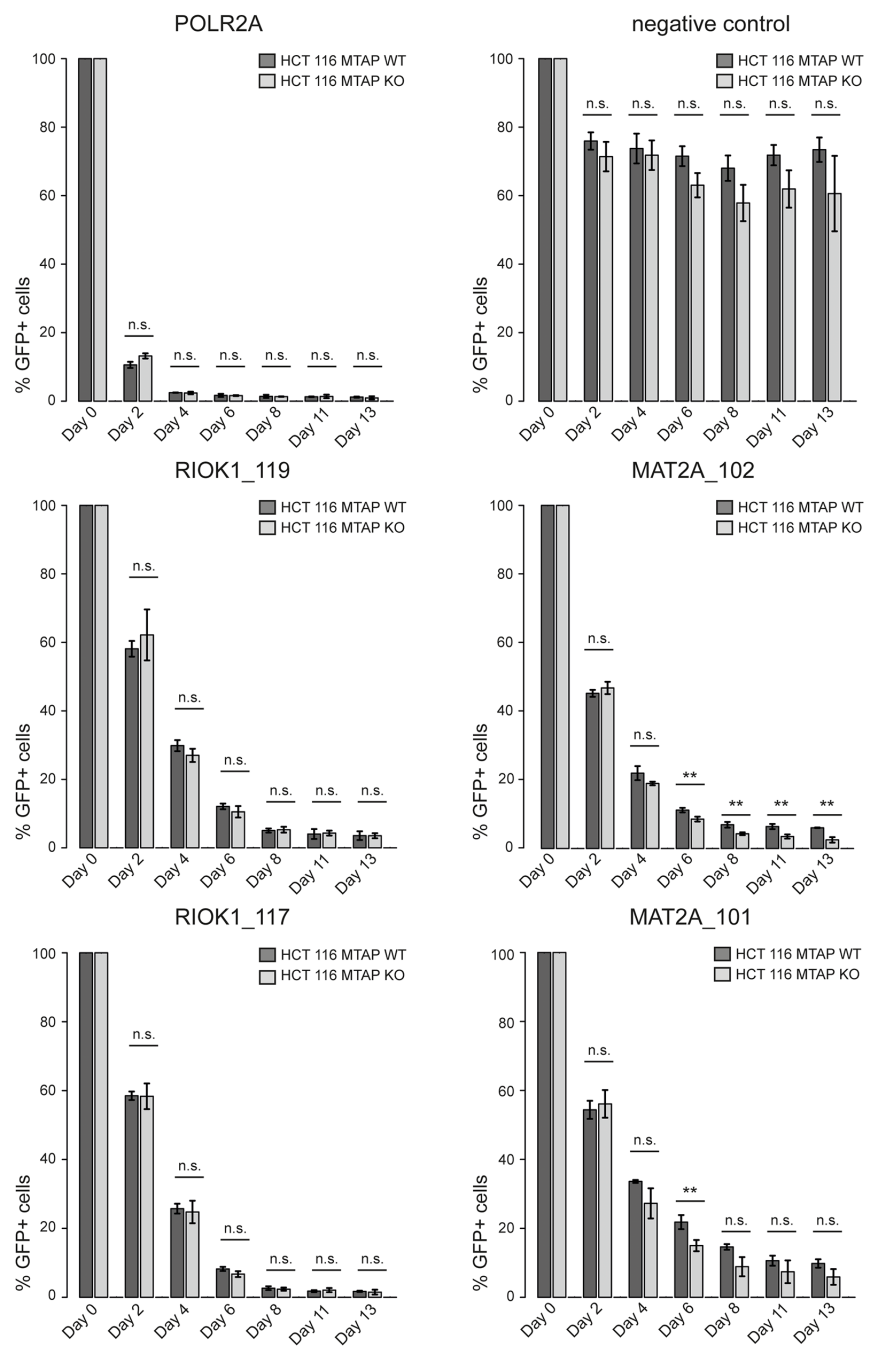
CRISPR based depletion assays reveal no differential requirement between MAT2A and RIOK1 in *MTAP* isogenic cell lines CRISPR based depletion time course experiments in HCT 116 *MTAP* isogenic cell lines. Each gRNA was tested in three independent biological replicates and mean and standard deviation (error bars) are plotted (n.s. indicates non-significant; ^**^ denotes p-values < 0.05; *t*-test). *POLR2A* serves as a core essential control gene. The ‘negative control’ refers to the same gRNA as used in Figure 2 (Neg.con._3). RIOK1_119, RIOK_117, MAT2A_102 and MAT2A_101 are representative gRNAs targeting *RIOK1* and *MAT2A* respectively.

As our CRISPR screens in *MTAP* isogenic cell lines represent an end point measurement after multiple population doublings, 13 for the kinase library screen and 18 for the epigenetic library, we wanted to determine if a difference in the depletion kinetics exists between *MTAP*-proficient and deficient cells. We reasoned that an acutely induced loss of PRMT5, RIOK1 or MAT2A function could result in differential depletion rates between *MTAP*-positive and negative cells. As PRMT5 is partially inhibited by the elevated MTA levels in *MTAP* null cells [5–7], further inhibition of PRMT5, RIOK1 or MAT2A function should result in an increased rate of cell death or a slower proliferation rate in a CRISPR depletion assay. We therefore designed gRNAs targeting PRMT5, MAT2A and RIOK1. After stable transduction of Cas9 into the isogenic HCT 116 *MTAP* cells, we infected cells with lentivirus particles co-expressing GFP and gRNAs targeting these genes. We followed the depletion of GFP-positive cells over time and found that targeting *PRMT5* in *MTAP* null cells resulted in a faster depletion of GFP-positive cells than in MTAP expressing cells. The maximum difference in the depletion of GFP positive cells was observed at day 6 (Figure 3A, 3B). Targeting *PRMT5* in *MTAP*-deleted HCT 116 cells with three representative gRNAs, resulted in a reduction in the fraction of GFP-positive cells to 31.2%, 28.89% and 27.46% respectively. In MTAP expressing HCT 116 cells however, the same gRNAs depleted the fraction of GFP-positive cells to 50.82%, 48.8% and 47.81%. Figure 3A, 3B and 3C summarize the observed depletion values for the individual gRNAs in our assay. These data reveal a small and transient difference in the depletion rates of *PRMT5* mutant cells between *MTAP*-proficient and deficient cells. To our surprise, under these conditions, no differential requirement was observed for gRNAs targeting *MAT2A* and *RIOK1* (Figure 4). gRNAs targeting *MAT2A* and *RIOK1* resulted in an almost complete depletion of GFP positive cells over 2 weeks with similar kinetics irrespective of *MTAP* status. Altogether, these data suggest that CRISPR/Cas9 based depletion assays can be used to probe a differential requirement of essential genes in different genetic backgrounds by quantifying a difference in the depletion kinetics.

### Pharmacological inhibition of RIOK1 and PRMT5 does not reveal a differential sensitivity between *MTAP* proficient and deficient cells

It has been proposed that inhibiting the enzymatic function of PRMT5 represents a therapeutic option to treat *MTAP* mutant cancers [5–7]. The PRMT5 inhibitor, EPZ015666 [23], does not recapitulate the enhanced genetic dependency of *MTAP*-deficient cells on PRMT5 function (Supplementary Figure 2A) (EC_50_ proliferation in MTAP wild type HCT 116 cells = 2.5 μM; EC_50_ proliferation MTAP knock out HCT 116 cells = 2.0 μM) [5–7]. At the concentrations tested, complete inhibition of symmetric arginine di-methylation in HCT 116 *MTAP* isogenic cells was observed at EPZ015666 concentrations >5μM (Supplementary Figure 2B). Consistent with previous results [5–7], global symmetric arginine di-methylation levels are reduced in *MTAP* deficient cells when compared to isogenic *MTAP* proficient cells (Supplementary Figure 2B). In addition to isogenic *MTAP* cell lines, we addressed differential sensitivity between *MTAP*-proficient and deficient cells towards PRMT5 inhibition in a panel of pancreatic cancer and non-transformed but immortalized cell lines that differ in *MTAP* status. We did not observe a differential sensitivity towards PRMT5 inhibition correlating with *MTAP* status (Supplementary Figure 2C). Of note, PRMT5 and RIOK1 protein levels are not altered upon changing *MTAP* status (Supplementary Figure 2D). Altogether, these data suggest that the functional status of MTAP does not induce a differential sensitivity towards PRMT5 inhibition.

A previous study demonstrated that, unlike wild type RIOK1, a K208R/D324N RIOK1 mutant that lacks catalytic activity cannot rescue the growth inhibition of *MTAP*-deficient cells following RIOK1 depletion [6]. These data raise the possibility that the RIOK1 kinase activity might be differentially required in *MTAP*-proficient and deficient cells but this hypothesis was not directly tested. As RIOK1 kinase activity constitutes a potential therapeutic target, we wanted to test if the kinase activity of RIOK1 is differentially required in *MTAP* isogenic cell lines using a chemical-genetic approach that allows pharmacological inhibition. We chose to pursue this question using analog sensitive kinase alleles [17, 24].

**Figure 5:**
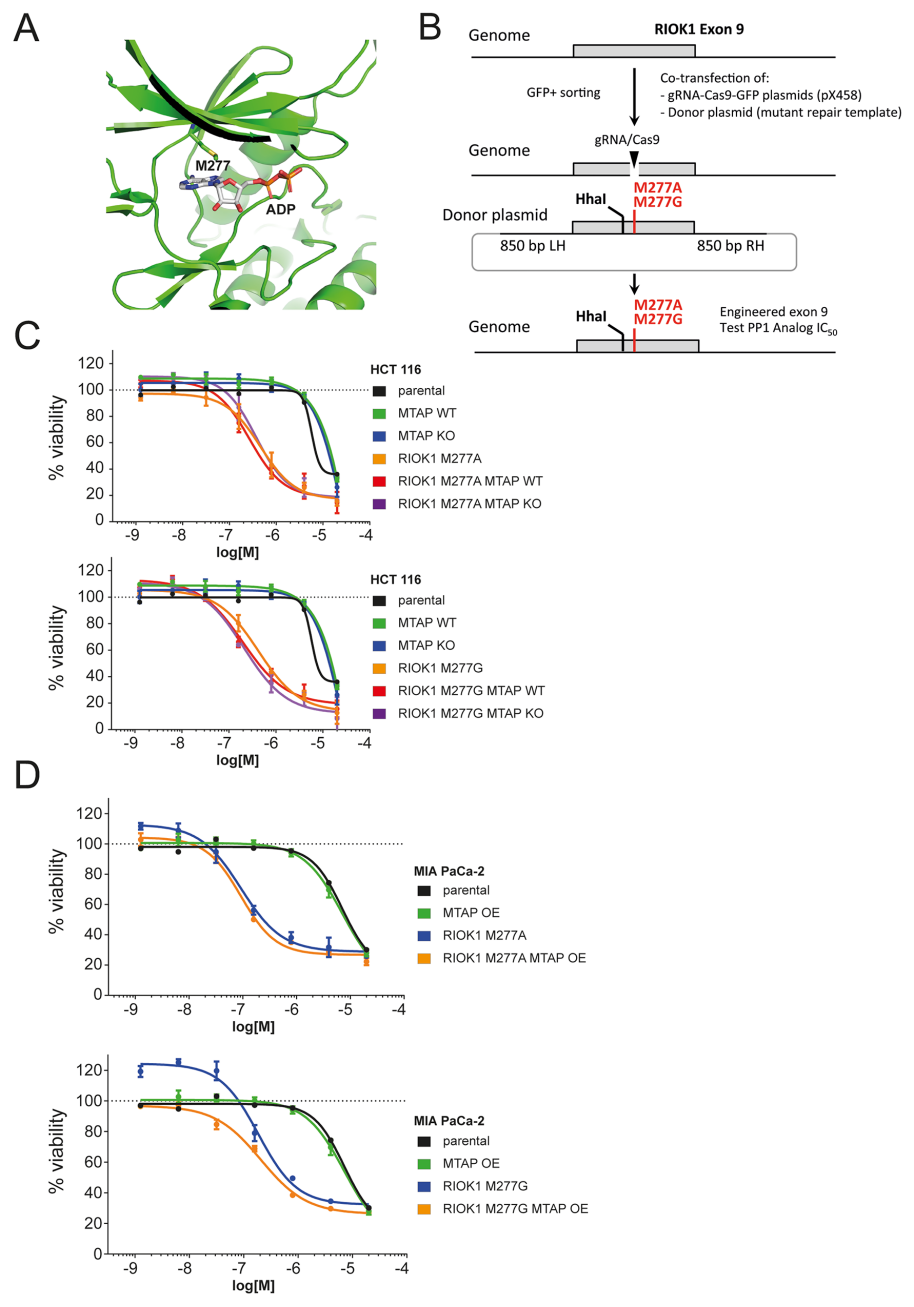
RIOK1 kinase activity is not differentially required in *MTAP*-proficient and deficient cells **(A)** Structural model of RIOK1 ATP binding pocket, bound to ADP, highlighting the gatekeeper residue M277. **(B)** Schematic representation of the CRISPR genome engineering strategy to introduce the M277A and M277G gatekeeper mutations in HCT 116 and MIA PaCa-2 cells. The restriction endonuclease site HhaI was introduced to facilitate PCR digest based identification of the edited allele. **(C)** Dose response curves of the 1-Naphtyl-PP1 (1-NA-PP1) analog in HCT 116 parental, M277A and M277G RIOK1 gatekeeper mutant cell lines in an *MTAP-*proficient or deficient background. **(D)** Dose response curves of the 1-NA-PP1 analog in MIA PaCa-2 parental, M277A and M277G RIOK1 gatekeeper mutant cell lines in an *MTAP*-proficient or deficient background.

Structural analysis of the RIOK1 kinase domain bound to ADP [25] revealed methionine 277 as the putative gatekeeper residue of RIOK1 (Figure 5A). We used CRISPR/Cas9 genome engineering and homologous recombination with a repair donor template to change the codon for M277 in exon 9 of *RIOK1* to either alanine or glycine in both HCT 116 and MIA PaCa-2 cells (Figure 5B, Supplementary Figure 3A–3D). Introduction of the gatekeeper mutation and/or deletion of *MTAP* do not impact the growth rate of the respective cells (Supplementary Figure 3B). Decreased levels of edited RIOK1 were observed (Supplementary Figure 3C), indicating potentially lower RIOK1 kinase activity levels in engineered cells. We subsequently tested a series of bulky ATP analogs and found that both gatekeeper mutations M277A and M277G confer strongly increased sensitivity to the analog 1-NA-PP1 (Supplementary Figure 3E) compared to *RIOK1* wild type cells (Figure 5C, 5D). Viability assays revealed that the introduction of the *RIOK1* mutations M277A and M277G causes a >10 fold sensitization of HCT 116 and MIA PaCa-2 cells to the bulky ATP analog 1-NA-PP1 (Figure 5C, 5D). These data suggest that alanine and glycine substitutions of methionine 277 result in an analog sensitive allele of *RIOK1* that is selectively inhibited by 1-NA-PP1. The viability decrease observed in analog sensitive allele carrying HCT 116 and MIA PaCa-2 cells strongly suggests that RIOK1 kinase activity is an essential function irrespective of the genetic background.

Having established analog sensitive *RIOK1* alleles, we wanted to test for the differential requirement of RIOK1 kinase activity in *MTAP-*proficient and deficient cells. We therefore generated *MTAP* isogenic cell lines in the *RIOK1* analog sensitive mutation backgrounds by disrupting the *MTAP* gene in HCT 116 cells and introducing an *MTAP* transgene into MIA PaCa-2 cells (Figure 1A). Next, we tested the impact of 1-NA-PP1 on *RIOK1* analog sensitive kinase cells with and without MTAP function. Importantly, the sensitivity RIOK1 analog sensitive HCT 116 and MIA Paca2 cells to 1-NA-PP1 was unaffected by altering the status of *MTAP*. Thus, no differential requirement for RIOK1 kinase activity was observed between *MTAP*-positive and negative isogenic HCT 116 and MIA PaCa-2 cell lines (Figure 5C, 5D).

We next wanted to determine if evidence for clone to clone variability can be detected in our assay. A larger panel of *MTAP* isogenic, RIOK1 analog sensitive allele carrying MIA PaCa-2 and HCT 116 cells was included in the assay. As shown in Supplementary Figure 4 (panels A and B), we obtained highly consistent EC_50_ values for the respective isogenic cell pairs. In addition to single drug treatments, we determined if combined inhibition of PRMT5 (by EPZ015666) and the analog-sensitive RIOK1 version by 1-NA-PP1 would synergize. Drug combination studies revealed no evidence for synergistic effects in RIOK1 M277A edited HCT 116 and MIA PaCa-2 cells, suggesting that no differential sensitivity exists between *MTAP*-proficient and deficient cells with respect to dual inhibition (Supplementary Figure 4C). Together, our data suggest that the kinase activity of RIOK1 is required for general cell survival and that this requirement is not enhanced by *MTAP* loss.

## DISCUSSION

It is the paramount goal of cancer drug discovery to identify therapeutics, that selectively impair the growth and survival of cancerous cells while sparing non-transformed cells. The concept of synthetic lethality in cancer refers to vulnerabilities that are associated with inherent differences between cancer and normal cells, generated in the course of neoplastic transformation. These differences can potentially be harnessed by cancer therapeutic approaches and should collectively be characterized by a superior therapeutic index when compared to conventional cytotoxic agents.

Three studies have independently reported an increased dependency of *MTAP*-deleted cells on components of a PRMT5 containing complex, including PRMT5, WDR77 and RIOK1 as well as the upstream factor MAT2A [5–7]. Due to the loss of *MTAP*, the upstream metabolite levels of MTA increase, which results in a partial inhibition of PRMT5. It has been suggested that this partial inhibition might render *MTAP*-deficient cells sensitive to further PRMT5 complex inhibition [5–7]. In our study, we have used additional experimental strategies, including time resolved CRISPR depletion assays, analog sensitive kinase mutants, CRISPR screens in MTAP isogenic cell lines and bioinformatics analysis of large scale CRISPR screens, to interrogate the proposed increased sensitivity of MTAP deleted cancer cells on the function of PRMT5, MAT2A and RIOK1. Our results confirm parts of the previous findings, but suggest that the window between *MTAP* null and wild type cells might be narrow and hard to exploit therapeutically: i) PRMT5 and its binding partners, WDR77 and RIOK1 as well as the upstream component MAT2A are core essential genes. We observed no or a very narrow and only transient window between *MTAP*-positive and negative cells in our PRMT5 CRISPR experiments. ii) The PRMT5 inhibitor EPZ015666 efficiently inhibits proliferation and the formation of symmetric di-methylarginines in *MTAP*-positive and negative isogenic cells at similar concentrations, further supporting the notion that the therapeutic window between *MTAP*-positive and negative cells is small. iii) Importantly, we were not able to detect a differential requirement of RIOK1 kinase activity in *MTAP* isogenic cell lines. We generated analog sensitive alleles of *RIOK1* [17] to probe its requirement in *MTAP*-positive and negative isogenic cells. These conditional alleles allowed us to demonstrate that *MTAP*-deficient HCT 116 and MIA PaCa-2 cells do not exhibit an enhanced sensitivity towards RIOK1 inhibition when compared to their isogenic *MTAP* wild type counterparts. Our data support the conclusion that RIOK1 kinase activity is generally required for cell survival irrespective of *MTAP* status. These data are consistent with a role of RIOK1 in ribosome biogenesis [25] which it presumably performs outside of its requirement in the complex with PRMT5. In line with these results, our CRISPR based experiments did not reveal an increased genetic dependency of *MTAP* null cells on RIOK1. Consistently, data published by Weinberg et al. suggest no differential sensitivity between different MTAP expressing and non-expressing cell lines to RIOK1 loss of function, although no isogenic cells were used in this study to directly test this hypothesis [26]. The experiments presented in this paper do not preclude the possibility that PRMT5, MAT2A or RIOK1 might be differentially required in cell lines that have larger deletions in the CDKN2A locus.

Despite our de-validation of MTAP as a biomarker, that predicts cellular sensitivity to RIOK1 inhibition, other molecular alterations of tumor cells might sensitize cancer cells to the inhibition of RIOK1 kinase activity. Weinberg et al. recently documented an increased requirement of RIOK1 in tumor cells with oncogenic Ras signaling [26] and these data suggest that RIOK1 is a potentially interesting target in *RAS* mutant tumors. In the context of *MTAP*-deleted cancers, our study de-validates RIOK1 kinase function as a target. *MTAP* status alone does not determine the sensitivity to RIOK1 inhibition and our data suggest that the kinase activity of RIOK1 is not a relevant therapeutic target for *MTAP*-deleted cancers. Finally, our work provides new tools and approaches to interrogate the cellular function of the poorly understood but essential kinase RIOK1 in human cells.

## MATERIALS AND METHODS

### Generation of *MTAP* isogenic HCT 116 and MIA PaCa-2 cell lines

HCT 116 and MIA PaCa-2 cells were cultured in McCoy´s 5A supplemented with 10% FCS in 12-well plates to achieve 20-30% confluency on the day of transfection. Cells were transfected with a plasmid ordered from SIGMA (U6gRNA-Cas9-2A-GFP, HS0000259922) encoding the Cas9 endonuclease, GFP and a MTAP targeting gRNA using X-tremeGENE 9 DNA transfection reagent (ROCHE #06356779001) according to the manufacturer’s protocol. 72 h after transfection individual GFP positive cells were sorted by FACS (SONY cell sorter S800Z) and seeded into 96-well plates for isolating single cell colonies. After 14 days of culture single cell-derived colonies were lysed directly in 96-well format using 4x Laemmli buffer + DTT, boiled for 5min at 95°C and analyzed by Western Blot.

### Antibodies used

Anti-MTAP, Abcam ab96231; anti-Actin, Sigma A5441; anti-SMDA, Cell Signaling 13222; anti-RIOK1, Abcam ab88496; anti-PRMT5, Abcam ab109451.

### Sequences

All gRNA, PCR primer and repair template sequences are listed in Supplementary Table 1.

### Generation of analog sensitive RIOK1 alleles

gRNAs targeting Exon 9 of the *RIOK1* locus were cloned into pX458 harboring the wild type Cas9 coding region, an gRNA expression cassette and a GFP reporter. The CRISPR construct and the respective RIOK1 M277A or M277G repair templates were transiently introduced into HCT 116 and MIA PaCa-2 cells in a 1:1 ratio. For transfection 2.5×10^5^ HCT 116 cells and 3.2×10^5^ MIA PaCa-2 cells were plated per well in a 6-well dish 24h prior transfection. HCT 116 cells were transfected using X-tremeGENE 9 DNA Transfection Reagent and MIA PaCa-2 using Lipofectamine 3000 Reagent. 48h after transfection, transduced cells were purified by FACS based on medium GFP expression. Subsequently, the cells were cultured for 5-7 days to allow genome editing and recovery. Recovered cells were plated into 96-well plates at single cell density. Subsequently, growing colonies were selected and DNA was isolated using QuickExtract™ DNA Extraction Solution. To detect effective genome engineered of the *RIOK1* alleles, PCR amplification of CRISPR/Cas9- induced genomic modification, restriction digest and Sanger Sequencing were performed.

### Metabolomics

Cell pellets of 10 million cells were washed in ice cold PBS and pellets were snap-frozen in liquid nitrogen and stored at -80°C until extraction. Cell pellets were subsequently extracted using a MeOH:ACN:H_2_O (2:2:1, v/v) solvent mixture. A volume of 1 mL of ice cold solvent was added to each pellet, vortexed for 30 s, and incubated in liquid nitrogen for 1 min. Subsequently, the sample was thawed at room temperature and sonicated. To precipitate proteins, the samples were incubated for 1 hour at −20°C, followed by 15 minutes centrifugation at 13,000 rpm and 4°C. The resulting supernatant was collected and evaporated to dryness @30°C in a vacuum concentrator. The dry extracts were then reconstituted in 100 μL of ACN:H2O (1:1, v/v), sonicated for 10 min, and centrifuged 15 min at 13000 rpm and 4°C to remove insoluble debris. The supernatants were transferred to HPLC vials and stored at −80°C prior to LC/MS analysis.

Extracted metabolites were diluted in 0.1% formic acid (FA) in water by mixing 50 μl of the sample with 50 μl 0.1% FA. 2 μl of the sample were injected and separated using a Ulitimate U300 BioRSLC HPLC system (Dionex; Thermo Fisher Scientific), employing a reversed phase column (Gemini,150 × 2 mm; 3 μm, 110 Å; Phenomenex). Separation was carried out with a flow rate of 100 μl/min using a linear gradient starting with 95% A (0.1% FA) to 60% B (acetonitrile, 0.1% FA) in 10 minutes, followed by re-equilibration of the column. Eluting metabolites were on-line analyzed using a TSQ Quantiva mass spectrometer (Thermo Fisher Scientific) after electrospray ionization with single reaction monitoring (SRM) in the positive ion mode using a spraying potential of 3500V. MTA was quantified using the transitions *m/z* 298 → 136 (with a collision energy (CE of 18)), SAM *m/z* 399 → 250 (CE14) and taurine *m/z* 126 → 108 (CE 10) and retention times of standard compounds were used for validation. Data were manually interpreted using the Xcalibur software (Thermo Fisher Scientific).

### Cell viability assays

24h prior analog treatment 1200 HCT-116 cells and 2500 MIA PaCa-2 cells were plated per well in a 96-well plate. The following analogs were tested: 1-NA-PP1 (Merck Millipore #529605), 1-NM-PP1 (Merck Millipore #529606), 3-IB-PP1 (Merck Millipore #529598), 3-MB-PP1 (Merck Millipore #529582) and 3-BrB-PP1 (Abcam #ab143756). Analog 1-NA-PP1 was added by using a HP D3000 Digital Dispenser. All treatments were performed in technical duplicates. Treated cells were incubated for 96h at 37°C with 5% CO_2_. CellTiter-Glo^®^ Luminescent Cell Viability Assay was performed and luminescence signal were detected by using the multilabel Plate Reader VICTOR X4. Quantifications of viable cells were calculated by normalization of analog treated cells to DMSO treated cells.

### CRISPR screens

Cas 9 expressing HCT 116 MTAP isogenic cell lines were infected with the indicated gRNA libraries at multiplicity of infection <1. Cells were grown for the respective cell doublings (indicated in main text). Subsequently, genomic DNA was extracted and amplicons around the gRNA were PCR amplified using primers shown in Supplementary Table 1. 50 ng of amplicons were subsequently used for library generation with the TruSeq Nano DNA Library Prep kit for NeoPrep (Illumina) and then sequenced on the HiSeq1500 in rapid mode with the paired end protocol for 50 cycles. A Python based script (available upon request) was used to count the individual gRNAs in the sequencing files. The CRISPR scores were calculated as [Plasmid read counts]/[screen read counts]. The log10 of this number is plotted in Figure 1C. The screening results are available in in Supplementary Tables 2–5. The individual gRNA sequences are available in Supplmentary Table 1.

### Bioinformatics analysis

Data from two large-scale CRISPR [20] and RNAi [21] screens were used. Cells were grouped according to MTAP expression using a cutoff of TPM < 2 as not expressed. Wilcoxon tests were performed for all genes screened in the respective studies, comparing MTAP expressing and non-expressing cells. For statistical tests, the *rsa* (RNAi study) and *ceres* (CRISPR study) scores were used as reported in the respective papers. P-values were corrected using a Benjamini-Hochberg procedure.

### Drug combinations

Drug combination studies were performed as previously described [27] using the Bliss Independence Model [28, 29].

### CRISPR-Cas9 experiments

An expression vector encoding Cas9 and a puromycin selection marker was stably integrated into the target cells of choice. Thereafter, cells were virally transduced with constructs encoding for GFP and gRNAs targeting *RIOK1*, *PRMT5* or *MAT2A*. The fraction of GFP positive cells was determined by FACS measurement. The fraction of GFP positive cells 2 days post infection was set to 100%.

## SUPPLEMENTARY MATERIALS FIGURES AND TABLES












