# Supplementary material for: RIOK1 kinase activity is required for cell survival irrespective of MTAP status
Source: Oncotarget. 2018 Jun 19;9(47):28625–37. doi: 10.18632/oncotarget.25586 (PMC6033344; doi:10.18632/oncotarget.25586)
Supplement: Supplementary file 1 [file oncotarget-09-28625-s001.pdf]

RIOK1 kinase activity is required for cell survival irrespective of *MTAP* status

SUPPLEMENTARY MATERIALS

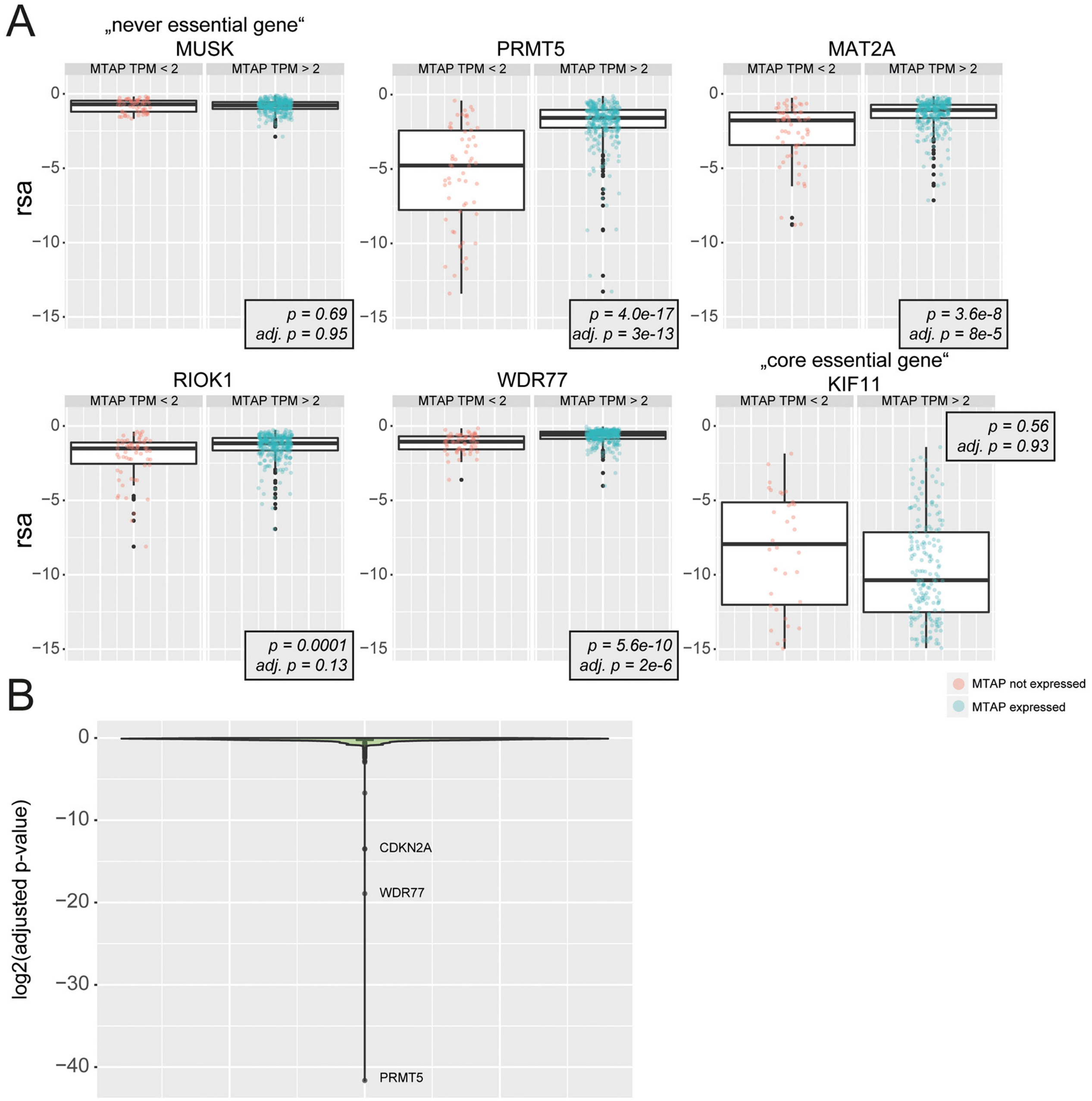

Supplementary Figure 1: (A) Boxplots (overlaid: individual data points) depict depletion scores between MTAP non-expressing cells (MTAP TPM < 2) and MTAP expressing cells (MTAP TPM > 2) for the "never essential gene" *MUSK, PRMT5, MAT2A, RIOK1, WRD77* and the "core essential gene" *KIF11*. P-values and adjusted p-values for Wilcoxon test statistics are indicated in boxes. Y- axis depicts the *rsa* score as reported in [21]. **(B)** Explorative analysis for differentially required genes between MTAP expressing and non-expressing cells. Violin- and boxplot of log2 adjusted p-values (Wilcoxon test).

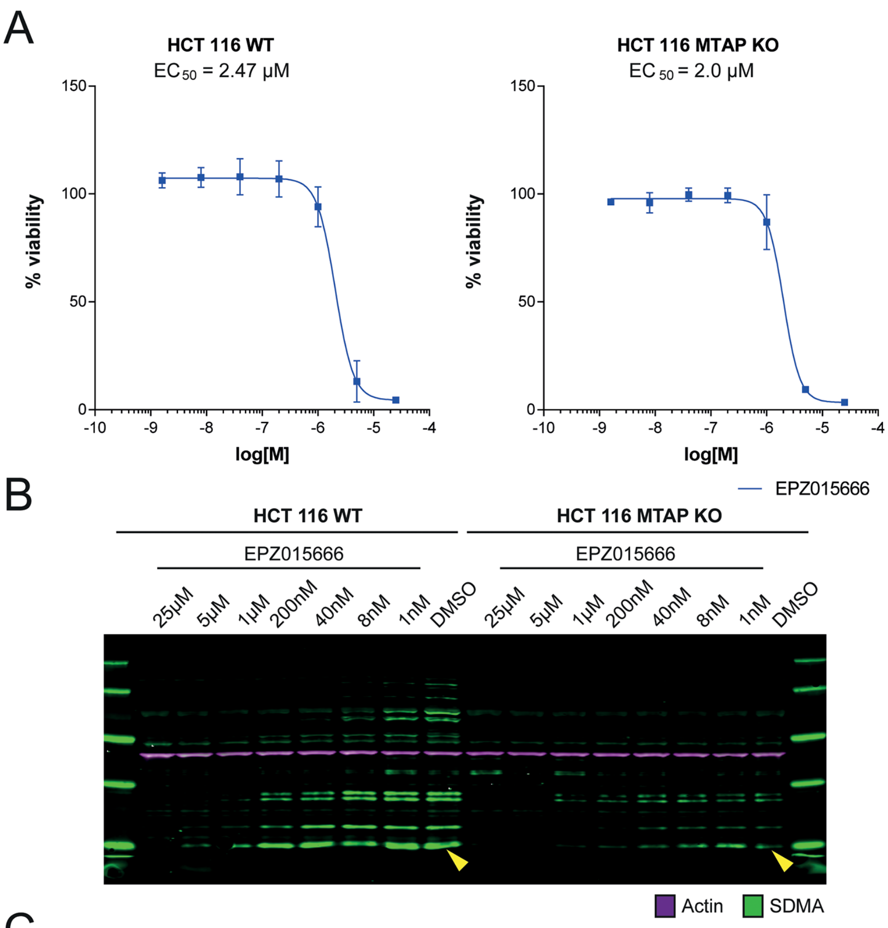

C

	MTAP TPM	EC_{50} [nM]
Pancreatic cancer cell lines		
**Capan-1**	1	4272
**MIA PaCa-2**	1	3955
**DAN-G**	1	6917
**KP-4**	1	4834
**BxPC-3**	1	>40000
**SU.86.86**	1	>40000
**QGP-1**	27	12060
**Panc 04.03**	61	11680
**KP-3**	78	>40000
**HPAF-II**	241	6643
**MIA PaCa-2 OE**	N.A.	12740
Immortalized/non-transformed cell lines		
**MCF 10A**	N.A.	1038
**h-TERT RPE-1**	50	>25000*

*Note: highest concentration measured: 25000 nM

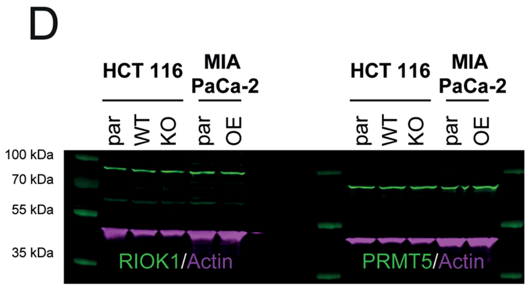

Supplementary Figure 2: (A) Dose response curves of EPZ015666 in HCT 116 *MTAP* WT and *MTAP* KO cells. **(B)** Western Blot for SDMA (green) and Actin (loading control, magenta) upon 96h treatment with different EPZ015666 concentrations. Yellow arrowhead points out comparable band at same size. **(C)** Cell viability assays performed after 8 days of incubation with EPZ015666. MTAP expression values are depicted in TPM (transcripts per million). EC_{50} values are in nM. Note: MIA PaCa-2 OE refers to MIA PaCa-2 cells overexpressing MTAP. TPM value for MIA PaCa-2 OE cell line has not been determined (N.A.). **(D)** Western blot assays comparing RIOK1 (green) and PRMT5 (green) protein levels in HCT 116 parental (par), HCT 116 wild type clone (WT), HCT 116 MTAP knockout (KO), MIA PaCa-2 parental (par) and MIA PaCa-2 MTAP overexpressing (OE) cells.

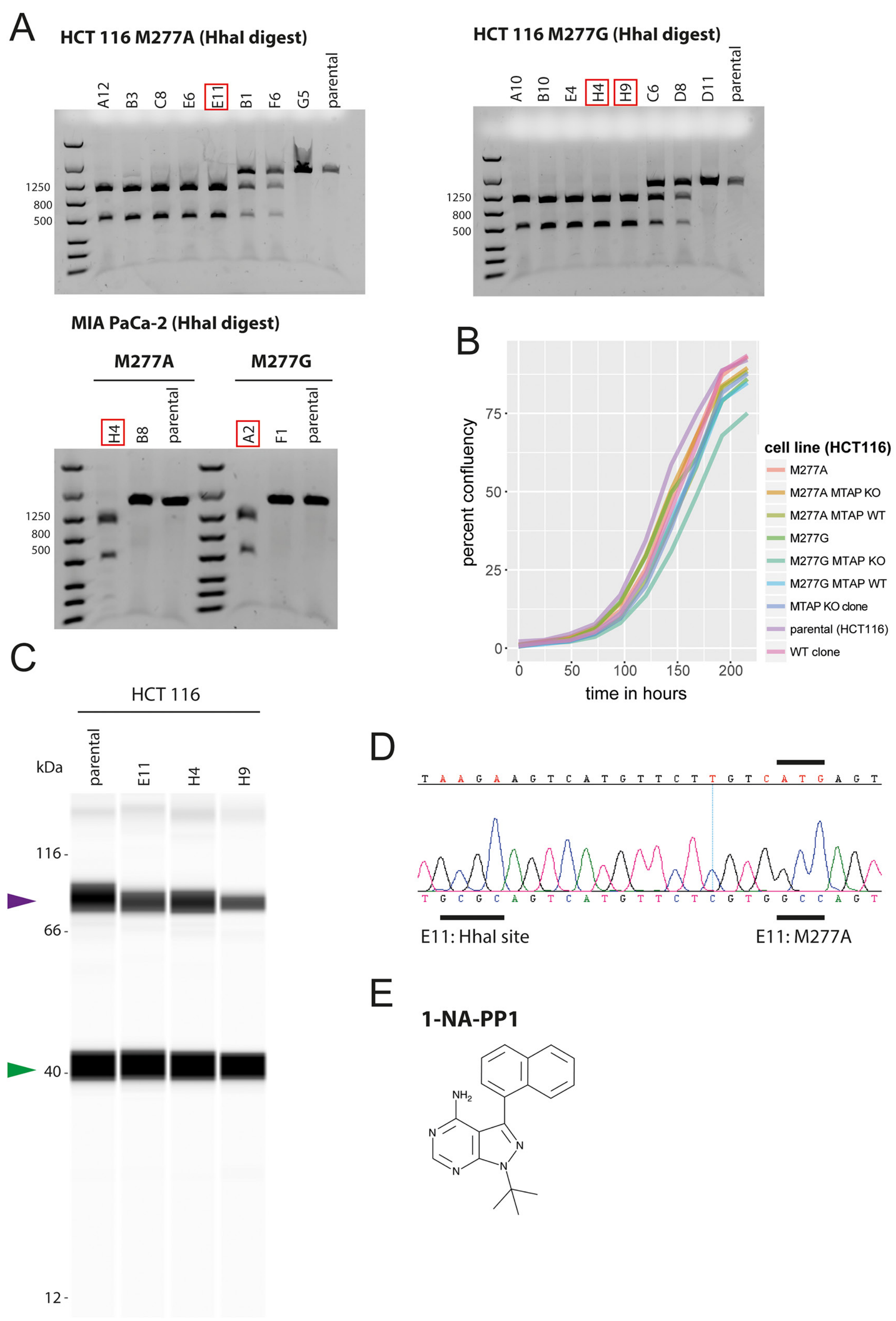

Supplementary Figure 3: (A) Restriction enzyme analysis of genome edited *RIOK1* alleles. Presence of double band indicates complete editing. Single uncut band indicates WT allele. Red boxes indicate selected clones. **(B)** Cell growth assay comparing the growth rate of different engineered and parental HCT 116 cell lines. **(C)** RIOK1 protein expression analysis using a capillary based immunoassay. Upper bands (magenta arrowhead) correspond to RIOK1; lower bands (green arrowhead) correspond to GAPDH. Cell lines and clone identifiers are indicated on top. **(D)** Representative chromatogram of a Sanger sequencing experiment to confirm the full editing of the RIOK1 gatekeeper residue of HCT 116 clone E11. **(E)** Structure of the 1-NA-PP1 analog.

A

MIA PaCa-2

	parental	MTAP OE
**MIA PaCa-2 parental**		
**1-NA-PP1**	8576.48	7984.05
**Panobinostat**	4.74	7.43
**M277A (H4)**		
**1-NA-PP1**	79.88	78.66
**Panobinostat**	3.82	4.19
**M277G (A2)**		
**1-NA-PP1**	234.52	379.28
**Panobinostat**	1.55	3.66

EC50 96h Cell Viability Assay [nM]

B

HCT 116

	parental	MTAP KO	MTAP WT
**HCT 116 parental**			
**1-NA-PP1**	4742.3	11434.3	12282.8
**Panobinostat**	9.2	8.0	7.9
**M277A (E11)**			
**1-NA-PP1**	461.89	270.2	373.0
**Panobinostat**	10.2	10.4	8.4
**M277G (H9)**			
**1-NA-PP1**	364.8	114.9	262.0
**Panobinostat**	10.0	7.3	7.8
**M277G (H4)**			
**1-NA-PP1**	579.3	360.3	568.2
**Panobinostat**	9.5	7.6	10.0

EC50 96h Cell Viability Assay [nM]

C

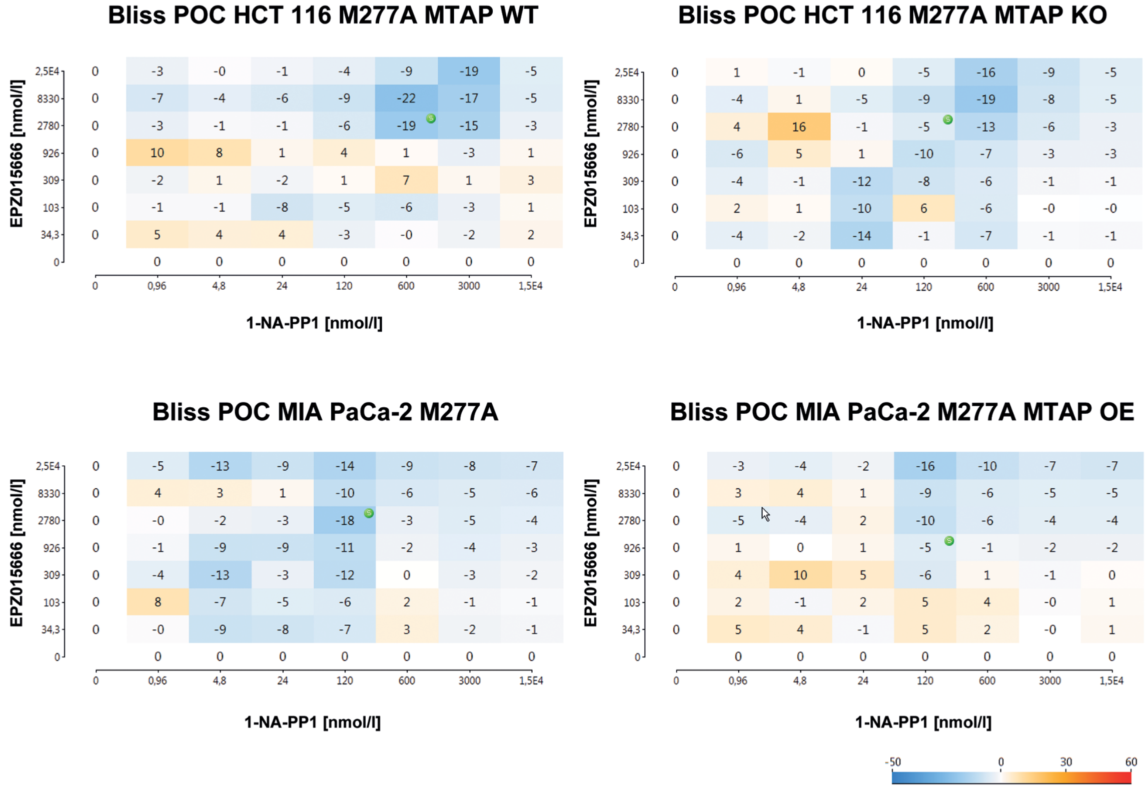

Supplementary Figure 4: (A) Table summarizing EC_{50} values of 1-NA-PP1 and Panobinostat (control) in multiple independently derived isogenic MIA PaCa-2 clones. **(B)** Table summarizing EC_{50} values of 1-NA-PP1 and Panobinostat (control) in multiple independently derived isogenic HCT 116 clones. **(C)** Combination drug matrix of percent of control (POC) CellTiter-Glo cell viability measurements of MIA PaCa-2 and HCT 116 *RIOK1* genome engineered cells treated with the indicated amounts of EPZ015666 and 1-NA-PP1. Values depict Bliss excess POC.

Supplementary Table 1: Sequences for gRNAs, primers and CRISPR genome engineering repair templates used in this study.

See Supplementary File 1

Supplementary Table 2: CRISPR screen results HCT 116 wild type cells - epigenome domain CRISPR library (gRNA information see supplementary table 1).

See Supplementary File 2

Supplementary Table 3: CRISPR screen results HCT 116 MTAP knock out cells - epigenome domain CRISPR library (gRNA information see supplementary table 1).

See Supplementary File 3

Supplementary Table 4: CRISPR screen results HCT 116 wild type cells - kinase domain CRISPR library (gRNA information see supplementary table 1).

See Supplementary File 4

Supplementary Table 5: CRISPR screen results HCT 116 MTAP knock out cells - kinase domain CRISPR library (gRNA information see supplementary table 1).

See Supplementary File 5
